# Editorial Policy

**Published:** 1918-08

**Authors:** 


// WAR MEDICINE

Published by the American Red Cross Society in France
for the
Medical Officers of the American Expeditionary Forces

**EDITORIAL OFFICES : 9, rue du Mont-Thabor, Paris (Ier)**

WAR MEDICINE *accepts no original articles. Its scope is limited to the publication of the proceedings of the Research Society of the American Red Cross in France, to abstracts of original articles, and to editorial comment on subjects pertaining to the medicine, surgery, and hygiene of the war.*

*Circulars, bulletins, and reports from the Office of the Chief Surgeon of the American Expeditionary Forces will also appear in* WAR MEDICINE.

WAR MEDICINE *is distributed free of charge to the medical officers of the American Expeditionary Forces.*

*All communications pertaining to the mailing list should be addressed to the Editorial Offices. If copies are not received promptly and regularly the managing editor should be informed at once. Addresses should be written distinctly.*

THE RESEARCH SOCIETY OF THE AMERICAN RED CROSS IN FRANCE
*All communications should be addressed to the Secretary,*
*9, rue du Mont-Thabor, Paris (1er)*

### EDITORIAL COMMENTS

A number of editorial notes and announcements have appeared in various numbers of the *Medical Bulletin*; but, up to this time, no department devoted to editorials has been deemed necessary. It seems, however, that with the natural development of the only medical publication for the rapidly growing American Expeditionary Forces in France, its educational effect may by enhanced by the judicious use of editorial comments.

As in the other departments of *War Medicine*, the editorial comments will be limited to subjects of interest to medical officers on duty with the American Expeditionary

Forces. The abstracts of original articles appearing in *War Medicine* endeavor to present, impartially, the authors' views; but it is often desirable to make a critical review of important contributions to medical literature, and a department for editorial comments appears to be the best means of meeting this need.

It has been suggested that since *War Medicine* is the official organ of the Research Society, editorial notes or comments on its meetings, giving impressions, or brief summaries, of some of the most important facts brought out in the discussions, would call attention to the fuller reports as published in the same number, and, by thus adding interest, would perbaps make them of greater practical value.

In order to make such editorial comments of authoritative value, those bearing upon subjects which come properly within the province of any of the regularly organized divisions, or sections, of the Medical Department of the American Expeditionary Forces, will be referred to medical officers in authority for review and comment. Furthermore, heads of divisions and those in charge of various activities of the American Expeditionary Forces will be requested to contribute editorial notes or comments on subjects related to their work. It is thus hoped to present many valuable and authoritative contributions, not intended in any sense to be official, on matters of vital importance in Army medical work.

The editorial comments in *War Medicine*, therefore, will not present merely the opinions of the editor, but will reflect the collective ideas of a number of men who desire to give medical officers the correct viewpoint on matters that will be helpful to them in performing their duties as Army surgeons.

